# Herbal Medicines Used for the Treatment of Diabetes Mellitus in Saudi Arabia

**DOI:** 10.7759/cureus.88497

**Published:** 2025-07-22

**Authors:** Osman Suliman, Zainab Abdullah A, Shatha AlKuhayli, Ruba N Alqahtani, Raghed Aljohani, Rahaf Almaimani

**Affiliations:** 1 Department of Clinical Sciences, Al-Rayan National College of Medicine, Al-Rayan National Colleges, Al-Madinah Al-Munawwarah, SAU

**Keywords:** complementary therapies, diabetes mellitus, herbal medicine, saudi arabia, therapies

## Abstract

Background

Due to the chronic nature of diabetes mellitus (DM) and the potential side effects associated with conventional medications, there has been growing interest in the use of complementary and alternative therapies for DM management.

Objective

The objective of this study was to assess the use of herbal medicines for the treatment of DM among diabetic patients in Saudi Arabia.

Methods

A cross-sectional study was conducted using a convenience sample of 53 diabetic patients (both genders, aged 20-55 years). A predesigned, structured questionnaire was used to collect data on patients’ demographics, DM-related clinical information, use of herbal products for DM treatment, types of herbs used, duration of use, perceived effectiveness in lowering blood sugar, any side effects, and sources of information about the herbs.

Results

Among the participants, 60.4% were aged 50-55 years, 79.2% were female, 54.7% had a university education, 45.3% were employed, 35.8% had a monthly income of 10,001-20,000 SAR, and 7.5% were ex-smokers. Chronic diseases were reported by 58.5% of participants, with hypertension (61.3%) and hypercholesterolemia (51.6%) being the most common comorbidities. The duration of DM was 16-20 years for 35.8% of participants, and 73.6% were on oral hypoglycemic agents. The prevalence of herbal medicine use was 56.6%, with cinnamon (43.4%), coriander (33.3%), and moringa (30%) being the most commonly used herbs. Most users (70.1%) reported that the herbal remedies were effective in lowering blood sugar levels, and none reported any side effects. No statistically significant associations were found between herbal medicine use and participants’ demographic or clinical characteristics.

Conclusions

More than half of the diabetic patients in this study reported using herbal remedies. Further longitudinal studies with larger sample sizes are needed to evaluate usage patterns and determine the potential benefits and/or risks of herbal medicine use in DM management.

## Introduction

One condition for which herbs are commonly used is diabetes mellitus (DM). Physicians treating diabetic patients must be aware of this fact and encourage open discussions about herbal use, as it may influence the course and effectiveness of conventional treatments [1].

A wide variety of plants are known to reduce glucose production, and herbal remedies have been employed in the treatment of insulin-dependent DM (IDDM), non-insulin-dependent DM (NIDDM), diabetic retinopathy, and diabetic peripheral neuropathy. Of the estimated 250,000 plant species, fewer than 1% have undergone pharmacological screening, and only a small proportion of these have been evaluated for their effectiveness against diabetes. Many widely used modern medications - such as aspirin, antimalarials, anticancer agents, and digitalis - originate from plants, making herbal medicine a logical area to explore for diabetes management. Based on current reports highlighting the potential antidiabetic effects of medicinal plants, it is reasonable to suggest that phytochemicals may play an important role in diabetes care [2].

Globally, over 300 million people are affected by DM, making it one of the most prevalent endocrine disorders. Allopathic treatments based on Western medicine can sometimes be ineffective, carry risks, or be too expensive, especially for those in developing countries. As such, the use of accessible, plant-derived compounds that do not require complex pharmaceutical manufacturing appears to be a promising approach to managing DM. Researchers in pharmacology and therapeutics are actively exploring evidence-based alternative medicines to treat various forms of diabetes in both humans and animals [3].

Herbal medicine typically relies on natural, plant-derived compounds readily available in the environment. Traditional herbal remedies have been effectively used to manage both type 1 and type 2 diabetes [4]. In recent years, herbal medicines have gained increasing attention as sources of hypoglycemic agents. The biological activity of these plant-based treatments depends on their chemical composition, which often includes phenolic compounds, terpenoids, flavonoids, coumarins, and other bioactive ingredients known to help lower blood sugar levels. Many herbal species have become popular for their antidiabetic properties because they are considered effective, associated with fewer side effects in clinical practice, and relatively affordable [5].

Given the widespread use of herbal remedies for diabetes, physicians should proactively engage their patients in conversations about these practices. Understanding the use of herbs is crucial, as it can significantly influence treatment outcomes [6]. Numerous plants have demonstrated the ability to reduce glucose synthesis, and herbal treatments have shown benefits for complications such as diabetic retinopathy, diabetic peripheral neuropathy, IDDM, and NIDDM. Despite this, less than 1% of the approximately 250,000 known plant species have been pharmacologically screened, and only a limited number have been evaluated for their potential in managing DM. Many essential modern drugs, including aspirin, digitalis, and various antimalarial and anticancer medications, are plant-derived. Therefore, it is logical to explore herbal options for diabetes treatment. Based on current literature, phytochemicals show considerable promise in the management of diabetes [7,8].

To validate traditional herbal practices, especially those used by rural populations, phytochemical and clinical research into these plants is strongly recommended. Identifying the active constituents responsible for their hypoglycemic effects should be a priority [9]. For example, in Guinea, herbal medicine is widely used for diabetes treatment. However, despite its popularity, there is limited scientific evidence supporting its efficacy. Patients should be made aware of potential side effects, and the conditions under which these remedies are used should be clearly defined [10-12]. Healthcare providers must also recognize that patients returning from overseas with so-called “herbal” treatments may be using substances that contain active pharmacological agents [11].

In Cameroon, herbal medicine is also a central part of diabetes management. Scientific validation has come through patient follow-up, which has documented effective glycemic control among individuals using family-based herbal remedies [13,14].

This study aimed to assess the use of herbal remedies for diabetes treatment among diabetic patients in Saudi Arabia.

## Materials and methods

Study design

A cross-sectional study was conducted in Saudi Arabia between May 2024 and April 2025.

Study participants

The inclusion criteria were diabetic patients of both genders aged between 20 and 55 years. Exclusion criteria included nondiabetic individuals, patients younger than 20 years, and those who declined to participate in the study.

Sampling technique

A convenience sampling technique was employed, and a total of 53 diabetic patients were included in the study.

Data collection

Data were collected using a predesigned, structured questionnaire. The questionnaire covered patients’ demographic information (gender, age, and education level) and clinical data, including type of diabetes, duration of diabetes, most recent HbA1c measurement, and prescribed medications. It also assessed the use of herbal products for diabetes management, types of herbs used, duration of use, perceived effectiveness in lowering blood sugar, any experienced side effects, and sources of information about the herbs.

Ethical considerations

The study protocol was approved by the Al Rayan Institutional Review Board. Participant confidentiality and privacy were ensured by encoding the data and removing all personally identifiable information.

Data analysis

Data were analyzed using IBM SPSS Statistics for Windows, Version 26.0 (Released 2018; IBM Corp., Armonk, NY, USA). Qualitative data were expressed as frequencies and percentages, and the chi-squared test (χ²) was used to examine associations between categorical variables. Quantitative data were presented as mean ± SD. A p-value of < 0.05 was considered statistically significant.

## Results

Of the 53 diabetic patients included in the study, 32 (60.4%) were aged between 50 and 55 years, and 42 (79.2%) were female. A total of 29 participants (54.7%) had attained a university level of education. Regarding employment status, 24 (45.3%) were employed, while 19 (35.8%) reported a monthly income between 10,001 and 20,000 Saudi Riyals. Four participants (7.5%) identified as ex-smokers. Additionally, 31 patients (58.5%) reported having a chronic condition other than diabetes, with hypertension (19 patients, 61.3%) and hypercholesterolemia (16 patients, 51.6%) being the most commonly reported comorbidities, as summarized in Table 1.

**Table 1:** Sociodemographic and clinical characteristics of the studied diabetic patients DM, diabetes mellitus; HTN, hypertension

Characteristic	Category	N	Percentage (%)
Age range	50-55 years	32	60.4%
Gender	Female	42	79.2%
Educational level	University	29	54.7%
Employment status	Employed	24	45.3%
Monthly income (Saudi Riyals)	10,001-20,000	19	35.8%
Smoking status	Ex-smoker	4	7.5%
Chronic disease (other than DM)	Present	31	58.5%
Common comorbidities	Hypertension	33	61.3%
	Hypercholesterolemia	27	51.6%

Among the 53 diabetic patients included in the study, the majority were female (42, 79.2%), and most were aged between 50 and 55 years (32, 60.4%). In terms of education, only one participant (1.9%) held a PhD, while the majority (29, 54.7%) had a university degree. The most common income range was 10,001-20,000 Saudi Riyals per month, reported by 19 participants (35.8%), and more than half (29, 54.7%) were unemployed. Regarding lifestyle factors, 49 participants (92.5%) were nonsmokers. Beyond DM, more than half (31, 58.5%) had at least one other chronic condition. The most commonly reported comorbidities were hypertension (19, 61.3%) and hypercholesterolemia (16, 51.6%), followed by hypothyroidism (7, 22.6%), cardiovascular disease (3, 9.7%), and asthma (3, 9.7%) (Table 2).

**Table 2:** Distribution of studied diabetic patients according to their demographic characteristics and chronic diseases CVD, cardiovascular disease; DM, diabetes mellitus; HTN, hypertension

Characteristic	Number of patients	Percentage (%)
Age (years)		
20-25	4	7.5%
26-30	3	5.7%
31-39	2	3.8%
40-49	12	22.6%
50-55	32	60.4%
Gender		
Female	42	79.2%
Male	11	20.8%
Educational level		
Reading and writing	3	5.7%
Secondary	18	34%
Diploma	2	3.8%
University	29	54.7%
PhD	1	1.9%
Occupational status		
Unemployed	29	54.7%
Employed	24	45.3%
Monthly income (Saudi Riyals)		
<5000	17	32.1%
5000-10,000	15	28.3%
10,001-20,000	19	35.8%
>20,000	2	3.8%
Smoking status		
Nonsmoker	49	92.5%
Ex-smoker	4	7.5%
Chronic diseases other than DM		
No	22	41.5%
Yes	31	58.5%
If having a chronic disease, specify: (no.: 31)		
Hypercholesterolemia	16	51.6%
HTN	19	61.3%
CVD	3	9.7%
Hypothyroidism	7	2.6%
Asthma	3	9.7%

Among the 53 diabetic patients studied, 56.6% (30 patients) reported using herbal remedies for diabetes management, while 43.4% (23 patients) did not use herbal treatments (Table 3).

**Table 3:** Prevalence of herbal remedy usage among diabetic patients

Herbal remedy usage	Number of patients	Percentage (%)
Used herbal remedies	30	56.6%
Did not use herbal remedies	23	43.4%

Among the 53 diabetic patients studied, 19 (35.8%) had a DM duration of 16-20 years, and 23 (43.4%) had their last HbA1c measurement more than three months ago. The majority, 45 (84.9%), were receiving medication for DM. Among those, 39 patients (73.6%) were taking oral hypoglycemic agents, as shown in Table 4.

**Table 4:** Clinical characteristics of the studied diabetic patients DM, diabetes mellitus; HbA1c, hemoglobin A1c

Characteristic	Number of patients	Percentage (%)
DM duration (years)		
<5	16	30.2%
5-10	16	30.2%
11-15	1	1.9%
16-20	19	35.8%
21-25	0	0.0%
26-30		1.9%
Last HbA1c measurement		
>3 months	23	43.4%
3 months	21	39.6%
1 month	9	17%
Are you taking medication for diabetes?		
No	8	15.1%
Yes	45	84.9%
DM medication type		
Oral hypoglycemic	39	73.6%
Insulin	14	26.4%

**Figure 1:**
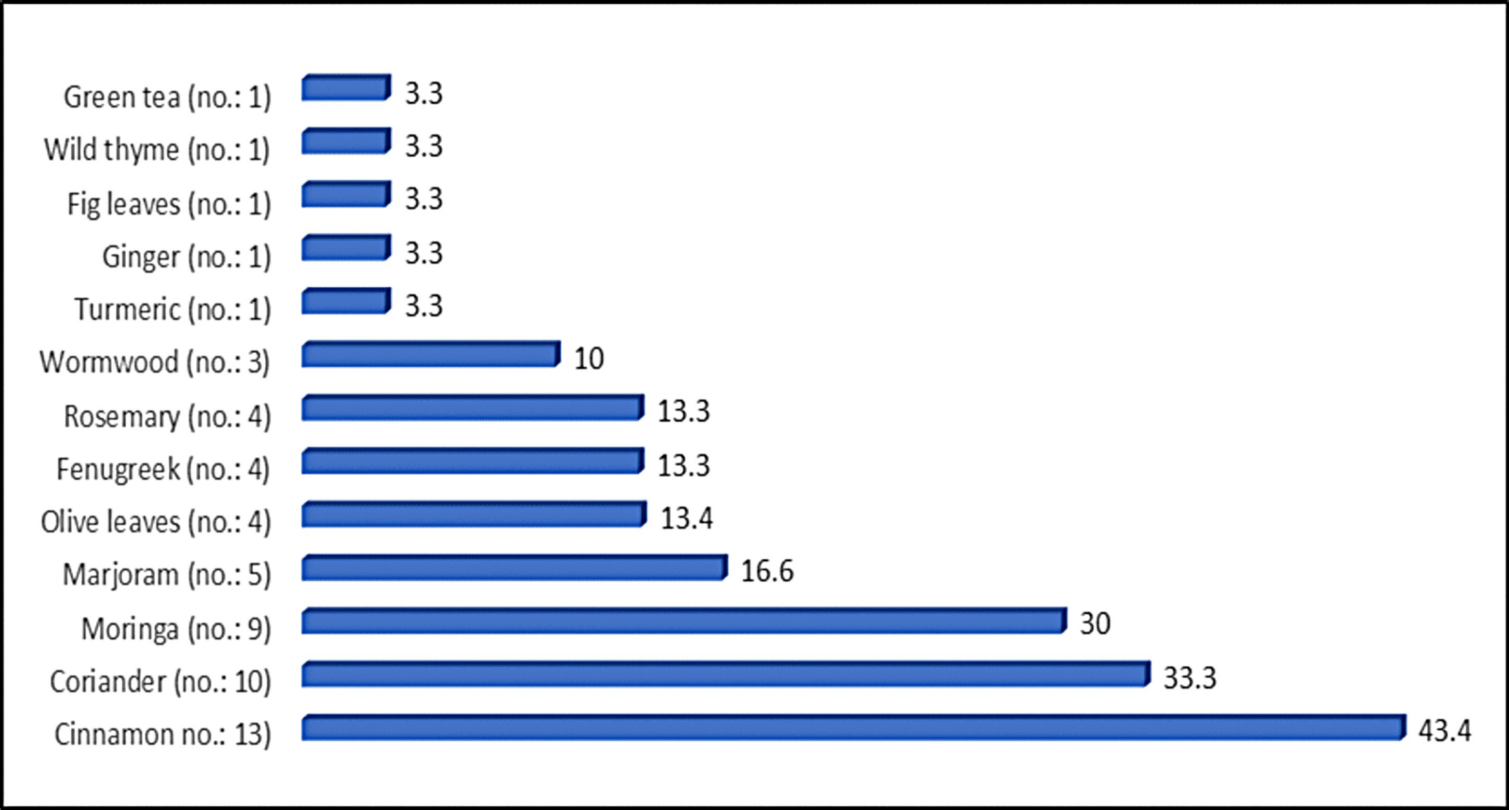
Distribution of herbal remedies used among diabetic patients

Among the 30 diabetic patients who reported using herbal remedies, the mean duration of herbal remedy use was 3.03 ± 3.86 years. The most commonly used herbs were cinnamon (13 patients, 43.4%), coriander (10 patients, 33.3%), and moringa (nine patients, 30.0%). The majority of users (21 patients, 70.1%) stated that the remedies were effective in lowering blood sugar levels, and none (0%) reported any side effects. Social media was the most frequently cited source of information on the benefits of these herbs, mentioned by 18 patients (33.4%) (Table 5, Figure 1).

**Table 5:** Patterns of herbal remedy usage, perceived effectiveness, and sources of information among diabetic patients

Characteristic	Number of patients	Percentage (%)
Herbal remedies use duration (years) (mean ± SD)	3	3.03 ± 3.86
Type of herbs		
Wormwood	3	10%
Coriander	10	33.3%
Cinnamon	13	43.4%
Fenugreek	4	13.3%
Moringa	9	30%
Marjoram	5	16.6%
Rosemary	4	13.3%
Olive leaves	4	13.3%
Turmeric	1	3.3%
Ginger	1	3.3%
Fig leaves	1	3.3%
Wild thyme	1	3.3%
Green tea	1	3.3%
Did you find it effective in lowering blood sugar?		
Maybe	7	23.3%
No	2	6.6%
Yes	21	70.1%
Did you find any side effects of the herbs?		
No	30	100%
Yes	0	0.0%
How did you know that this herb is beneficial for diabetics?		
Family members	10	33.3%
Social media	18	33.4%
Friends	10	33.3%

The Y-axis lists the specific herbal remedies used by participants, with the number of users indicated in parentheses. The X-axis shows the percentage of users who reported each herb. Cinnamon was the most frequently used remedy (43.4%), followed by coriander (33.3%), moringa (30%), and marjoram (16.6%). Olive leaves, fenugreek, and rosemary were each reported by approximately 13.3-13.4% of participants. Other herbs - such as wormwood, turmeric, ginger, fig leaves, wild thyme, and green tea - were reported less frequently, as illustrated in Figure 1.

According to responses from the 30 diabetic patients who used herbal remedies, 16 (53.4%) believed that their overall health had been positively impacted. However, most reported no noticeable changes in energy levels or fatigue (25, 83.4%), mood or emotional health (27, 90.0%), ability to perform daily tasks (27, 90.0%), physical activity levels (27, 90.0%), or capacity to manage their daily schedule (26, 86.7%) (p < 0.05, Table 6).

**Table 6:** Perceived impact of herbal remedy use on health and daily activities among diabetic patients

Characteristic	No (n, %)	Yes (n, %)
Have you felt that your overall health has improved as a result of using herbal remedies for diabetes?	14 (46.6%)	16 (53.4%)
Have you experienced an improvement in your energy levels or a reduction in fatigue since using herbal remedies?	25 (83.4%)	5 (16.6%)
Have you noticed any improvement in your mood or emotional health since using herbal remedies?	27 (90%)	3 (10%)
Have you found that using herbal remedies has improved your ability to perform daily activities, such as work or housework?	27 (90%)	3 (10%)
Have you experienced any changes in your ability to engage in physical activity or exercise since using herbal remedies?	27 (90%)	3 (10%)
Have you noticed any changes in your ability to manage your daily routine and schedule since using herbal remedies?	26 (86.7%)	4 (13.3%)

Table 7 shows that herbal remedy use was more prevalent among older participants aged 50-55 years (19, 63.3%), females (23, 76.7%), those with a university education (17, 56.7%), and employed individuals (16, 53.3%). However, none of these associations reached statistical significance (p > 0.05). Similarly, higher usage was observed among participants with a monthly income of 10,001-20,000 SR (13, 43.3%), nonsmokers (28, 93.3%), and those with comorbid chronic diseases (16, 53.3%), but these differences were also not statistically significant (p > 0.05).

**Table 7:** Association between herbal remedy use and sociodemographic and clinical characteristics among diabetic patients CVD, cardiovascular disease; DM, diabetes mellitus; HTN, hypertension

Characteristic	Herbal remedies use				χ²	p-Value
	No		Yes			
	No.	Percentage (%)	No.	Percentage (%)		
Age (years)						
20-25	3	13%	1	3.3%	7.66	0.105
26-30	3	13%	0	0.0%		
31-39	0	0.0%	2	6.7%		
40-49	4	17.4%	8	26.7%		
50-55	13	56.5%	19	63.3%		
Gender						
Female	19	82.6%	23	76.7%	0.27	0.597
Male	4	17.4%	7	23.3%		
Educational level						
Reading and writing	2	8.7%	1	3.3%	1.52	0.823
Secondary	8	34.8%	10	33.3%		
Diploma	1	4.3%	1	3.3%		
University	12	52.2%	17	56.7%		
PhD	0	0.0%	1	3.3%		
Occupational status						
Unemployed	15	65.2%	14	46.7%	0.18	0.179
Employed	8	34.8%	16	53.3%		
Monthly income (Saudi Riyals)						
<5000	9	39.1%	8	26.7%	1.81	0.612
5000-10,000	7	30.4%	8	26.7%		
10,001-20,000	6	26.1%	13	43.3%		
>20,000	1	4.3%	1	3.3%		
Smoking status						
Nonsmoker	21	91.3%	28	93.3%	0.07	0.782
Ex-smoker	2	8.7%	2	6.7%		
Chronic diseases other than DM						
No	8	34.8%	14	46.7%	0.75	0.384
Yes	15	65.2%	16	53.3%		
If having a chronic disease, specify: (no.: 31)						
Hypercholesterolemia	8	53.3%	8	50.0%	0.03	0.853
HTN	10	66.7%	9	56.3%	0.35	0.552
CVD	0	0.0%	2	18.8%	3.11	0.078
Hypothyroidism	3	2%	4	25.0%	0.11	0.739
Asthma	2	13.3%	1	6.30%	0.44	0.505

Table 8 shows no statistically significant association between herbal remedy use and clinical characteristics of diabetes. Nonetheless, higher usage was noted among patients with a DM duration of less than five years (11, 36.7%), those who had an HbA1c test in the last three months (14, 46.7%), those currently receiving diabetes medications (23, 76.7%), and those using oral hypoglycemics (24, 80.0%). These associations did not reach statistical significance (p ≥ 0.05).

**Table 8:** Association between herbal remedy use and clinical characteristics of diabetic patients DM, diabetes mellitus; HbA1c, hemoglobin A1c

Characteristic	Herbal remedies use				χ²	p-Value
	No		Yes			
	No.	Percentage (%)	No.	Percentage (%)		
DM duration (years)						
<5	5	21.7%	11	36.7%	3.43	0.487
5-10	8	34.8%	8	26.7%		
11-15	1	4.3%	0	0.0%		
16-20	9	39.1%	10	33.3%		
21-25	0	0.0%	0	0.0%		
26-30	0	0.0%	1	3.3%		
Last HbA1c measurement						
>3 months	11	47.8%	12	40.0%	1.59	0.451
3 months	7	30.4%	14	46.7%		
1 month	5	21.7%	4	13.3%		
Are you taking medication for diabetes?						
No	1	4.3%	7	23.3%	3.66	0.056
Yes	22	95.7%	23	76.7%		
DM medication type						
Oral hypoglycemics	15	65.2%	24	80.0%	1.46	0.226
Insulin	8	34.8%	6	20.0%		

## Discussion

The purpose of this study was to evaluate the common forms and perceived benefits of using herbal medicines for the treatment of DM in Saudi Arabia. In the current study, 56.6% of the diabetic patients reported using herbal remedies. This aligns with a prior Saudi study by Yaghmour et al. (2023), which found that 40.9% of 1290 diabetic patients used complementary and alternative medicine (CAM) [15]. A descriptive review of 36 national surveys conducted in Saudi Arabia between 2000 and 2015 reported a wide range of CAM use, varying from 21.6% to 90.5% [16].

A 2016 descriptive review by Erku and Mekuria (2016) found that CAM use in Saudi Arabia ranged from 17.4% to 64%, with herbs and honey being the most common forms [17]. In Taif, 33.7% of diabetic patients were reported to use CAM [18], while in Al-Ahsa, Al-Luwaym et al. (2024) reported a prevalence of 21.2% among diabetic patients [19].

A 2021 systematic review and meta-analysis of 38 studies found that the prevalence of CAM use among diabetic patients ranged from 8% to 89%, with an overall prevalence of 51% [20]. These findings are consistent with a prior comprehensive review of 18 studies from nine countries, which reported a prevalence range of 17.2% to 72.8% [21]. The current study’s CAM usage rate was lower than that reported in a Malaysian study (62.5%), while Bahrain showed a higher prevalence compared to other Gulf countries [22]. These differences may be attributed to variations in study design, cultural perceptions of CAM, accessibility and availability of remedies, and the types of CAM used.

In this survey, the most commonly used herbal remedies were cinnamon (43.4%), coriander (33.3%), and moringa (30%). Al-Luwaym et al. (2024) reported fenugreek (48.1%), cinnamon (45.3%), and black seed (27.4%) as the most frequently used remedies in Eastern Saudi Arabia [19]. Another Saudi study found that ginger (55.1%), cinnamon (64.6%), and bitter apple (95.3%) were the most common [15]. Similarly, Abdullah et al. (2020) reported bitter apple, garlic, fenugreek, ginger, and cinnamon as the top remedies [18].

In the current study, none of the participants reported side effects, and the majority (70.1%) believed the remedies were effective in lowering blood sugar. In Taif, 49.2% of respondents rated CAM as very effective, while 72.9% reported no side effects; 47.5% stated they would recommend CAM to others [18]. In a 2023 Saudi study, 38.8% of participants reported improved psychological well-being from CAM use, although 39.3% found it to have limited value [15]. These findings are consistent with international research that demonstrates high levels of satisfaction with CAM use among diabetic patients [23].

In this study, no statistically significant association was found between herbal remedy use and participants' demographic characteristics. However, greater usage was observed among older adults, females, those with higher education, those who were employed, those who had higher income, those who were nonsmokers, and those who had chronic comorbidities. In Al-Ahsa, the most significant factors associated with CAM use were older age, diabetes-related complications, and infrequent physician visits [19]. Yaghmour et al. also identified associations with older age, female gender, marital status, use of hypoglycemics, dyslipidemia, chronic illness, family history of diabetes, and HbA1c levels between 7% and 10% [15].

Several Saudi studies have shown that women are more likely to use CAM, with notable gender-based differences in usage [16]. Abdullah et al. (2020) found that female gender, longer DM duration, family history of DM, and complications were significantly associated with increased CAM use [18]. This higher prevalence of CAM use among women mirrors findings from international studies, which also report a strong correlation between female gender and CAM utilization [24].

Limitations

The study has several limitations. The use of a self-reported questionnaire may introduce recall bias. Additionally, the small sample size and cross-sectional design limit the ability to establish causal relationships and generalize the findings to the wider diabetic population.

## Conclusions

This study found that 56.6% of diabetic patients used herbal remedies, with cinnamon, coriander, and moringa being the most commonly used. These findings highlight the need for larger longitudinal studies to better understand usage trends, potential therapeutic benefits, and safety concerns associated with herbal remedies. Developing targeted interventions and integrating evidence-based herbal treatments into the healthcare system may help optimize patient care and promote open provider-patient communication regarding herbal medicine use.

## Appendices

**Table 9:** Study questionnaire and response options

No.	Question	Response options
1	Gender	Female, Male
2	Age	20-25 years, 25-30 years, 30-39 years, 40-49 years, ≥50 years
3	Education level	Read and write, High school, University
4	Occupation	Employed, Unemployed
5	Monthly income	<5000 SAR, 5000-10,000 SAR, 10,000-20,000 SAR, >20,000 SAR
6	Smoking	Nonsmoker, Former smoker, Active smoker
7	Comorbidity	Yes, No
8	Type of comorbidity	Cardiovascular disease, Diabetes mellitus, Hypothyroidism, Hypertension, Asthma, High cholesterol, Rheumatoid disease
9	Diabetic patient	Yes, No
10	Type (if diabetic)	Prediabetic, Type 1, Type 2, Gestational diabetes
11	Since when have you had diabetes?	>5 years, 5-10 years, 10-15 years, 15-20 years, More
12	Last HbA1c	(Open-ended)
13	Any side effects of diabetes	(Open-ended)
14	Have you ever taken herbs for diabetes	Yes, No
15	Name of herbs	(Open-ended)
16	Effective in reducing blood sugar	Yes, No
17	Side effects of herbs	(Open-ended)
18	For how long have you used herbs?	(Open-ended)
19	How did you know this herb is beneficial?	Family, Friends, Social media
